# Successful pulmonary vein isolation and left atrial roofline ablation via jugular access using a novel cryoballoon to treat persistent symptomatic atrial fibrillation in a patient with congenital interruption of the inferior vena cava

**DOI:** 10.1016/j.hrcr.2025.07.023

**Published:** 2025-07-31

**Authors:** Yumi Yasui, Yuichiro Sagawa, Osamu Inaba, Hideki Arima, Tetsuo Sasano, Yasuteru Yamauchi

**Affiliations:** 1 Department of Cardiology, Japan Red Cross Yokohama City Bay Hospital, Shin-Yamashita, Yokohama, Japan; 2 Department of Cardiology, Japanese Red Cross Saitama Hospital, Saitama, Japan; 3 Department of Cardiovascular Medicine, Institute of Science Tokyo, Tokyo, Japan

**Keywords:** Azygous vein, Cryoballoon ablation, Persistent atrial fibrillation, Jugular approach, Interrupted inferior vena cava

**Key Teaching Points**

• Pulmonary vein isolation (PVI) and left atrial (LA) roofline ablation were successfully performed using the POLARx™ cryoballoon via the right internal jugular vein in a patient with interrupted inferior vena cava, demonstrating the feasibility and safety of superior venous access.
• The POLARx system achieved effective PVI and sufficient nadir temperature drop despite incomplete occlusion, owing to its compliant balloon design, its flexible sheath, and the availability of 2 balloon sizes.
• LA roofline ablation using this approach achieved isolation of >80% of the posterior wall, which may contribute to improved rhythm outcomes in patients with persistent atrial fibrillation.

## Introduction

Catheter ablation has become an established treatment for atrial fibrillation (AF), with pulmonary vein isolation (PVI) serving as the cornerstone of the procedure. Typically, the left atrium (LA) is accessed via the femoral vein and inferior vena cava (IVC). However, in rare congenital anomalies such as IVC interruption with azygos continuation, this route is not feasible, necessitating an alternative superior approach through the jugular vein and superior vena cava (SVC). This approach presents unique technical challenges, particularly for transseptal puncture and stable catheter manipulation. Recent advances in cryoballoon technology, such as the POLARx FIT system (Boston Scientific), offer improved flexibility and cooling performance, potentially enabling effective ablation in anatomically complex cases. Here, we report the first successful case of PVI and LA roofline ablation using the POLARx FIT cryoballoon via the jugular vein approach in a patient with IVC interruption, highlighting the feasibility and clinical value of this strategy in challenging anatomical scenarios.

## Case report

A 68-year-old male patient with a chief complaint of palpitations was diagnosed with paroxysmal AF. Three years later, his condition progressed to persistent AF, and he was referred to our hospital for catheter ablation. The use of antiarrhythmic drugs was contraindicated because the patient also had sick sinus syndrome. Echocardiography revealed a left ventricular ejection fraction of 61% and an enlarged LA (diameter, 50 mm). Initial ablation was performed, during which we discovered that the patient had an IVC anomaly wherein the vessel merged into the SVC via the azygos vein, making the LA approach challenging. Consequently, only a cavotricuspid isthmus block was created during the first ablation. Despite the subsequent administration of antiarrhythmic drugs, the sinus rhythm was not restored, and the patient’s palpitations persisted. Therefore, a second session was attempted using an SVC approach.

**Figure 1:**
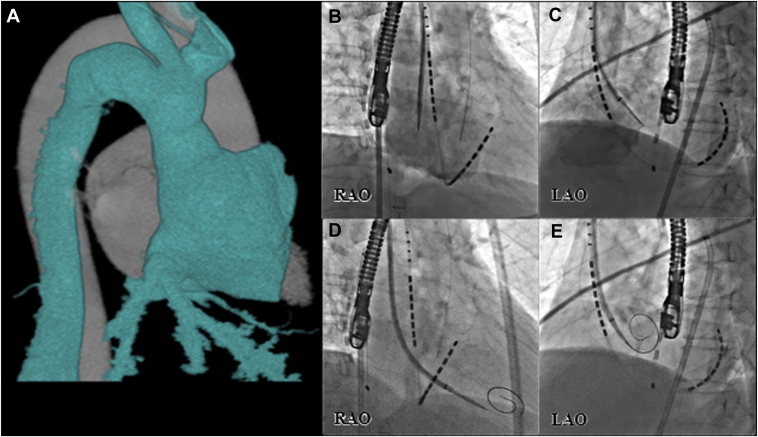
Contrast-enhanced computed tomography and transseptal puncture via the right internal jugular vein. **(A)** Contrast-enhanced computed tomography (RAO view) showing congenital interruption of the inferior vena cava with azygos vein continuation. **(B–E)** Fluoroscopic images illustrating the step-by-step process of transseptal puncture via the right internal jugular vein.**(B, C)** The sheath was positioned at the fossa ovalis under guidance via right atrial angiography and transesophageal echocardiography (RAO and LAO views). **(D, E)** The radiofrequency wire was advanced into the left atrium, and the spiral was deployed, facilitating sheath passage across the septum. LAO, left anterior oblique; RAO, right anterior oblique.

Contrast-enhanced computed tomography (CT) revealed the absence of the IVC, with the abdominal venous return draining into the right atrium via the hemiazygos vein and SVC (Figure 1). All of the patient’s PVs were considered anatomically suitable for cryoballoon ablation.

After obtaining written informed consent, the ablation procedure was performed under general anesthesia with propofol and endotracheal intubation, while the patient was receiving dabigatran. No sedation-related complications were observed with the internal jugular approach.

Transseptal puncture was performed by targeting the center of the fossa ovalis guided by fluoroscopy and transesophageal echocardiography using the SupraCross® radiofrequency (RF) system (Baylis Medical) (Figure 1). Santangeli^1^ previously reported successful transseptal puncture using this system via the SVC approach for AF ablation. The SupraCross sheath is a unidirectional deflectable sheath with a larger curve, flexible dilator, and smoother transition between the dilator and sheath, which allows the needle to obtain stable and vertical contact on the fossa ovalis. In our case, we used an ultrasound-guided short-axis approach to puncture the distal right internal jugular vein, thereby minimizing the risk of arterial injury. Transseptal puncture was performed using the specialized pigtail RF wire of the system, which has an exposed metal tip at its distal end to deliver a short burst of RF energy for the puncture. This wire significantly simplifies the puncture technique without the need for wire exchanges. Following a successful transseptal puncture on the first attempt, heparin was administered to maintain an activated clotting time of >350 seconds during the procedure. On the morning of the procedure, the patient’s anticoagulation regimen was switched from edoxaban to dabigatran. A 7-F multipolar catheter (EsoMeter Catheter; Japan Lifeline, Japan) was inserted into the esophagus to monitor esophageal temperature.

Three-dimensional CT images of the LA and PV were merged using an electro-anatomical mapping system (CARTO; Biosense Webster). A 48-electrode OctaRay catheter (Biosense Webster) was used to perform high-density voltage mapping. We then exchanged the SupraCross sheath for a POLARx sheath (Boston Scientific).

**Figure 2:**
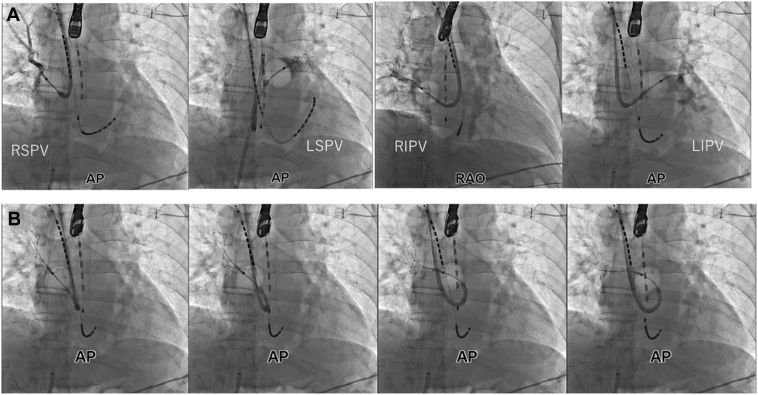
Cryoballoon ablation of the pulmonary vein and left atrial roof via the right internal jugular vein. **(A)** Fluoroscopic views of cryoballoon ablation for all pulmonary veins (PVs). Complete PV occlusion was achieved in all PVs except the left inferior PV. Left interior PV isolation was successfully accomplished using a separate freezing technique, with a sufficient nadir temperature drop despite incomplete occlusion. **(B)** Fluoroscopic views of cryoballoon ablation along the LA roof. During LA roofline ablation, the nadir temperature consistently reached below −40°C, thus facilitating the successful creation of a roofline block. A 31-mm balloon was used for the RSPV and LSPV, and a 28-mm balloon was used for the RIPV, LIPV, and the LA roofline. AP = anteroposterior; LA = left atrium; LSPV = left superior pulmonary vein; RAO = right anterior oblique; RIPV = right inferior pulmonary vein; RSPV = right superior pulmonary vein.

PVI was performed using the POLARx FIT cryoballoon (Boston Scientific). A POLARx map catheter was inserted via the cryoballoon catheter to monitor the PV potentials. The positioning of the cryoballoon during applications for each PV is illustrated in Figure 2.

The sheath exhibited sufficient flexibility, enabling it to adhere closely to the balloon (Figure 2). In the left superior PV, we obtained complete occlusion using a 31-mm balloon. The time to isolation was 109 seconds, and a single 240-second application of cryothermal energy was delivered, achieving a minimal temperature of –64°C. Complete occlusion could not be achieved in the left inferior PV (LIPV); hence, the separate freezing technique was applied for the superior and inferior portions of the LIPV, resulting in successful LIPV isolation. In the right superior and inferior PVs, we obtained complete occlusion using 31- and 28-mm balloons, respectively. A 28-mm cryoballoon was also used to perform LA roofline ablation (Figure 2B), using the same technique via femoral access. Ablation near the esophagus, including the left superior PV, LIPV, and LA roofline, was performed with sheath rotation to increase the distance from the esophagus. Each application was terminated if the esophageal temperature dropped below 15°C.

**Figure 3:**
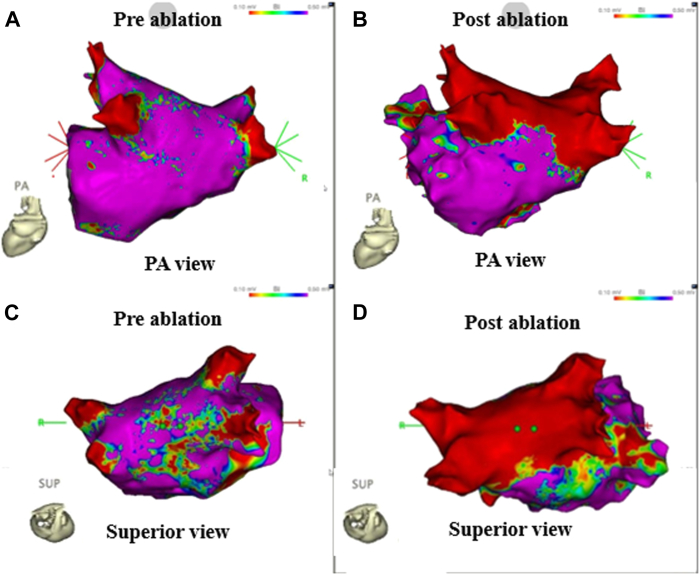
High-density bipolar voltage maps acquired using the CARTO 3 system, before and after cryoballoon ablation. **(A, C)** Pre-ablation voltage maps, posteroanterior (PA) and superior views. **(B, D)** Post-ablation voltage maps under the same views, confirming successful pulmonary vein isolation and roofline ablation. Over 80% of the left atrial posterior wall was isolated, as indicated by the extensive low-voltage area (*red*).

The time required to achieve PVI with the cryoballoon was 46 minutes. The total time required for PVI and LA roof ablation using the cryoballoon was 68 minutes. Post-ablation high-density voltage mapping confirmed the successful completion of both PVI and LA roofline ablation, with >80% of the LA posterior wall being isolated (Figure 3). Hemostasis following removal of the POLARx sheath was achieved by manual compression combined with a Z-suture. Protamine was administered to reverse heparin, and no closure device was used.

There were no postoperative complications. No arterial injury, hematoma, airway compression, or vagal reflexes occurred during the procedure. The patient has maintained sinus rhythm for 12 months following the ablation.

## Discussion

Congenital IVC interruption is a rare anomaly. A previous report noted IVC interruption in 8 (0.1%) of 7972 patients.^2^

Previous reports have documented 3 cases of PVI performed using the Arctic Front Advance cryoballoon via the jugular vein.^3^, ^4^, ^5^ However, none of these cases involved LA roofline ablation. Compared with previous reports using the Arctic Front Advance cryoballoon, PVI via the SVC approach using the POLARx system has achieved better PV occlusion and lower nadir temperatures. These improvements may be attributed to the enhanced flexibility of the POLARSHEATH system and specific characteristics of the POLARx FIT cryoballoon, including its lower internal pressure, compliant design, and availability of 2 adjustable balloon sizes.

Shigeta et al^6^ reported that the nadir cryoballoon temperature of POLARx system was approximately 10°C lower than that of the AFA-Pro system, consistently dropping below −40°C during LA roofline ablation using a cryoballoon. The POLARx cryoballoon can achieve sufficient nadir temperatures even under nonocclusive conditions.

Santangeli^1^ reported 15 cases of PVI with RF ablation via the SVC approach, 5 of which involved persistent AF. LA posterior wall isolation was performed in 1 case, but the procedure time was 462 minutes. PVI and LA posterior wall isolation via the SVC approach is challenging with RF ablation.

Kuniss et al^7^ reported that LA roofline ablation in addition to PVI, using the AFA-Pro cryoballoon, improved long-term outcomes compared with PVI alone, without increasing the complication rate. These findings suggest that LA roofline ablation may represent a valuable adjunctive strategy for patients with persistent AF undergoing cryoballoon ablation. Additionally, Shigeta et al^6^ demonstrated that, compared with the AFA-Pro system, LA roofline ablation using a cryoballoon is easier to perform and more reliable using the POLARx system.

For these reasons, we selected POLARx cryoablation for this case of persistent AF in a patient with LA dilation. Creating an LA roofline using the POLARx system resulted in the isolation of >80% of the LA posterior wall area, effectively achieving an outcome comparable with LA posterior wall isolation. This probably contributed to the favorable clinical outcomes we subsequently noted.

In our experience, roofline ablation was technically easier via the jugular approach, as the catheter could be advanced along a more direct and stable trajectory along the LA roof, similar to the ease of Swan-Ganz catheter insertion via the jugular vein. Additionally, the POLARSHEATH is highly flexible and can bend up to 180 degrees, which facilitates stable contact with the LA roof and allows effective lesion delivery. A previous study comparing the nadir temperatures of the POLARx and AFA-Pro Advance systems reported that the POLARx achieved PVI at an average temperature over 8°C lower than the AFA-Pro. During non-occlusive ablation of the LA roofline, the POLARx reached temperatures approximately 10°C lower. It has also been reported that achieving a temperature below –37°C is necessary to create a complete block line along the LA roof. These findings suggest that the POLARx system, with its ability to reach significantly lower temperatures, may offer advantages for effective LA roofline ablation. The combination of a superior access route and the unique features of the POLARx system enabled effective PVI and roofline ablation in this patient with an interrupted IVC. While similar outcomes may be achievable with other cryoballoon systems, the enhanced flexibility and performance of the POLARx system under non-occlusive conditions may be particularly beneficial in complex anatomies. In addition to rare congenital anomalies such as IVC interruption, this technique may also apply to patients in whom femoral venous access is contraindicated or technically challenging. Such scenarios include individuals with chronic lower limb deep vein thrombosis (DVT) or those with a permanent IVC filter, both of which are more commonly encountered in clinical practice. In these cases, a superior approach via the jugular vein, combined with the enhanced flexibility of the POLARx system, may provide a safe and effective alternative for performing AF ablation.

## Conclusion

To our knowledge, this is the first reported case of successful PVI and LA roofline ablation using the novel POLARx FIT cryoballoon via the right jugular vein approach. Cryoballoon ablation using the POLARx FIT system appears to be a safe and effective strategy when a superior approach via the SVC is required.

## Funding Sources

This research did not receive any specific grant from funding agencies in the public, commercial, or not-for-profit sectors.

## Disclosures

The authors have no conflicts of interest to disclose.
